# Direct comparison of canine and human immune responses using transcriptomic and functional analyses

**DOI:** 10.1038/s41598-023-50340-9

**Published:** 2024-01-26

**Authors:** Lyndah Chow, William Wheat, Dominique Ramirez, Renata Impastato, Steven Dow

**Affiliations:** 1 Flint Animal Cancer Center, Department of Clinical Sciences and Department of Microbiology, Immunology, and Pathology, College of Veterinary Medicine and Biomedical Sciences, Colorado State University, Campus Delivery 1678, Fort Collins, CO USA; 2 Department of Biochemistry, University of Colorado Boulder, Boulder, CO USA

**Keywords:** Immunotherapy, Lymphocytes, Translational immunology

## Abstract

The canine spontaneous cancer model is increasingly utilized to evaluate new combined cancer immunotherapy approaches. While the major leukocyte subsets and phenotypes are closely related in dogs and humans, the functionality of T cells and antigen presenting cells in the two species has not been previously compared in detail. Such information would be important in interpreting immune response data and evaluating the potential toxicities of new cancer immunotherapies in dogs. To address this question, we used in vitro assays to compare the transcriptomic, cytokine, and proliferative responses of activated canine and human T cells, and also compared responses in activated macrophages. Transcriptomic analysis following T cell activation revealed shared expression of 515 significantly upregulated genes and 360 significantly downregulated immune genes. Pathway analysis identified 33 immune pathways shared between canine and human activated T cells, along with 34 immune pathways that were unique to each species. Activated human T cells exhibited a marked Th1 bias, whereas canine T cells were transcriptionally less active overall. Despite similar proliferative responses to activation, canine T cells produced significantly less IFN-γ than human T cells. Moreover, canine macrophages were significantly more responsive to activation by IFN-γ than human macrophages, as reflected by co-stimulatory molecule expression and TNF-α production. Thus, these studies revealed overall broad similarity in responses to immune activation between dogs and humans, but also uncovered important key quantitative and qualitative differences, particularly with respect to T cell responses, that should be considered in designing and evaluating cancer immunotherapy studies in dogs.

## Introduction

Large animal cancer models that more effectively recapitulate the human immune response to novel immunotherapies or to novel immunotherapy combinations are needed to advance the cancer immunotherapy field. The value of the canine spontaneous cancer model has been recognized for years, and recently this has resulted in allocation of substantial NIH support for canine immunotherapy studies^1^. Particularly valuable are the canine cancer models, as the dog cancers more closely recapitulate human tumor biological behavior, genetics, histology, and/or response to therapy. Notable dog cancer models that have published clinical trial and mechanistic data include osteosarcoma, brain cancer, B cell lymphoma, melanoma, sinonasal carcinoma, and invasive uroepithelial cancers^2–4^.

There are several important examples of immunotherapy drugs being advanced by early trials in dogs with cancer. The biological response modifier L-MTP (liposomal muramyl tripeptide) was extensively evaluated in dogs with metastatic osteosarcoma, and these studies were instrumental in the eventual approval of the drug in Europe^5,6^. More recently, the dog glioma model has been used to evaluate tumor vaccines combined with checkpoint inhibitors, to assess responses to virolytic therapies, and to determine responses to TME (tumor microenvironment) modifying immunotherapies^7–9^

Our group has recently reported results from clinical trials using the dog osteosarcoma and brain cancer models to investigate the impact of repurposed immunotherapy drug combinations on tumor responses^8,10^; which show a clinical benefit rate of up to 80% in canine glioma and a clinical benefit rate of 50% in osteosarcoma dogs with lung metastases. Studies in the canine uroepithelial cancer model identified unique driver mutations such as BRAF mutations and have demonstrated target engagement with molecularly targeted drugs^11–13^. An early generation tyrosine kinase inhibitor for dogs (toceranib) was developed in parallel with sunitinib for similar cancers^14^. The first approved cancer vaccine in veterinary medicine was developed for dogs with melanoma, validating an approach that was initially developed in rodent models^15^. Thus, there are numerous examples of early and influential immunotherapy studies conducted in dogs.

Despite the long history of cancer immunotherapy development facilitated by studies in dogs with their spontaneous cancers, to date there have not to our knowledge been any comprehensive studies comparing human and canine immune responses directly. It is known for example that the major immune cell populations are similar in abundance between dogs and humans^16,17^, but their functionality has not been directly compared. Therefore, the current study was designed to provide a direct comparison of the functionality of canine and human T cells and macrophages, using in vitro activation protocols, and applying transcriptomic analysis coupled with conventional immune response assays.

Using RNA sequencing, we investigated how canine and human T cells responded to activation in vitro, to better understand their similarities and differences. Cytokine assays were then used to validate some of the findings from the RNA sequencing studies. Overall, we concluded that human and canine immune responses to activation were broadly similar, with conservation of key immune pathways. However, important differences were also discovered, particularly with respect to the functional bias of CD4 T cell pathways and the expression of certain T cell activation genes. The studies described here therefore provide important new context for understanding and interpreting immunological studies in dogs as a cancer immunology and immunotherapy model.

## Materials and methods

### Blood collection and peripheral blood mononuclear cell (PBMC) processing

Blood samples were collected by jugular vein venipuncture from n = 10 healthy dogs ranging in age from 2 to 10 years, with n = 4 female dogs and n = 6 male dogs. All dogs were determined to be free from medical conditions by physical examination and routine screening blood work. Breeds included an Australian Shepard dog, a Standard Poodle, a Pit Bull, and several mixed breed dogs. These studies were approved by the Colorado State University Institutional Animal Care and Use Committee and the CSU Institutional Review Board. The animal studies were performed and outcomes were reported were in accordance with ARRIVE guidelines (https://arriveguidelines.org). All methods were performed in accordance with the relevant guidelines and regulations'. For studies of human leukocyte responses, blood samples were collected by peripheral vein venipuncture from n = 10 healthy human volunteers, ranging in age from 22 to 65 years. There were n = 4 females and n = 6 males in the study population, and samples were obtained from volunteers of mixed ethnicity. Informed consent was obtained from all individual human participants included in the study. The human sample collection was approved by the CSU Institutional Review Board and Human Research Protection Program (HRPP). Results published using human subjects contain no information that could lead to the identification of a participant. Research involving human research participants was performed in accordance with the Declaration of Helsinki-ethical principles for medical research involving human subjects. Informed consent was obtained from all participants. The EDTA anti-coagulated blood samples were processed within 1 h of collection, using Ficoll density gradient centrifugation and Ficoll-Paque® PLUS separation solution (VWR, Radnor, PA), according to manufacturer’s instructions. All PBMCs used in this study showed > 70% viability after Ficoll separation. All blood samples and cytokine and sequence data obtained from blood samples from human volunteers were de-identified during all steps of processing and data analysis.

### PBMC culture and T cell activation

Following Ficoll separation, PBMC for both species were washed with PBS, counted and added to 24-well plates in triplicate cultures, at a cell density of 2 X 10^6^ cells per mL in complete culture medium. Complete cell culture medium consisted of DMEM supplemented with amino acids (essential and non-essential), 1% penicillin–streptomycin, Glutamax (ThermoFisher Scientific, Waltham, MA) and 10% fetal bovine serum (Peak Serum, Inc, Wellington, CO). For T cell activation, phytohemagglutinin (PHA) (Sigma-Aldrich, St. Louis, MO) was added to cultures at a concentration of 5 ug/ml for 72 h (cytokine and proliferation measurement) or for 24h (RNA sequencing). In some studies, cells were activated non-specifically using phorbol myristate acetate (PMA) at 50 ng/mL (Sigma-Aldrich, St. Louis, MO) plus ionomycin at 1ug/mL (Sigma-Aldrich, St. Louis, MO) for 5h in complete medium. Cells were incubated in a 5% CO2 incubator (ThermoFisher) at 37 °C.

### Measurement of secreted IFN-γ by ELISA

Supernatants from PHA treated or untreated cultures were collected after 72h in culture and stored at − 80 °C prior to analysis. Concentrations of IFN-γ, were assessed using species specific cytokine ELISA assays (R&D Systems), according to manufacturer guidelines. Human sample supernatants were diluted 1:3 in culture media to conform to the limit of detection. Cell culture media was used for background correction. Undiluted unstimulated samples from both canine and human cultures were used in ELISA. Below background OD (optical density) levels are represented as zeros on figures.

### T cell proliferation measured by EdU incorporation

Cultures of PBMC at density of 2 X 10^6^ cells/ml in complete medium were stimulated for 72 h with PHA (5 ug/ml) in 96 well flat bottom plates (Corning Inc. Corning, NY). Cell proliferation was assessed by adding 5-ethynyl-2'-deoxyuridine (EdU) (ThermoFisher Scientific) at a concentration of 0.01 mM, which was added 24 h after the cells were initially activated with PHA. Non adherent cells were collected After 48h hours of additional culture, and EdU incorporation was measured using Click-iT™ Plus Alexa Fluor™ 647 Flow Cytometry Assay Kit (ThermoFisher Scientific), following manufacturer’s instructions. To analyze T cell proliferation, cells were stained with an anti-CD5 FITC conjugated antibody for canine T cells (rat anti dog clone YKIX322.3, ThermoFisher Scientific) or an FITC conjugated anti-CD3 antibody (mouse anti human clone UCHT1, ThermoFisher Scientific) for human T cells. Flow cytometry was performed as previously described^18^. Cells were analyzed on a Beckman-Coulter Gallios flow cytometer. Cell analysis was done using FlowJo v9 software (BD Life Sciences, Franklin Lakes, NJ). Examples of FlowJo gating schemes for human and canine raw dot plots are shown in (Supplementary Fig. 1).

### Measurement of intracellular IFN γ production by T cells following activation by PMA and ionomycin

To assess the T cell response to activation by a second non-specific mitogen, PBMC were stimulated using PMA (50 ng/mL) and ionomycin (1ug/mL) (Sigma-Aldrich, St. Louis, MO). Monensin (1uM) (Sigma-Aldrich) was added to the cultures to retain secreted cytokines intracellularly. Cells were cultured for 5 h, then non adherent cells were collected, fixed and immunostained using the eBioscience™ Intracellular Fixation & Permeabilization Buffer Set (ThermoFisher Scientific) following the manufacture’s two-step protocol for detection of intracellular proteins. Cells were also immunostained with anti-canine CD5 or anti-human CD3 antibodies to identify T cells. To detect intracellular IFNγ production in canine T cells, a mouse anti-bovine IFNγ-RPE antibody^19^ (clone CC302, Biorad Laboratories, Hercules, CA) was used. Intracellular IFNγ production by human T cells was measured using an anti-IFN-γ- APC antibody mAb clone 4S.B3 (ThermoFisher Scientific). Appropriate irrelevant control antibodies included mouse IgG1-RPE(Biorad) for canine samples or mouse IgG1-APC (ThermoFisher Scientific) for human cells. Cells were analyzed using a Beckman-Coulter Gallios Flow Cytometer and data was analyzed using FlowJo v9. The fold change (FC) of geometric mean fluorescence intensity (gMFI) was calculated by determining the percentage of change in MFI divided by the untreated cell MFI.

### Monocyte-derived macrophage cultures

To generate monocyte-derived macrophage (MDM) cultures, PBMC were added to triplicate wells of 24-well plates (Corning Inc,) at a concentration of 4 × 10^6^ cells per mL and allowed to adhere for 4h, after which non-adherent cells were removed by gentle washing with PBS. Next, the adherent cells were cultured in complete cell culture medium (see above) to which was added 20 ng/ml hu M-CSF (PeproTech, Inc. Cranbury, NJ) to promote monocyte differentiation into macrophages. Medium was changed every 2–3 days and fresh M-CSF was added until MDM differentiation was complete at 7 days in culture, as reported previously^20^

### Measurement of MHCII and CD86 expression by IFNγ -treated macrophages

Macrophages were treated for 36 h with human (Preprotech, Cranbury, NJ) or canine (R & D Systems, Minneapolis, MN) species-specific recombinant IFN-γ, at concentrations ranging from 1 pg/ml to 1 μg/mL. The macrophages were then detached using ice-cold 3 mM EDTA (Sigma-Aldrich), after which the cells were immunostained with FITC-conjugated anti-human HLA-DR (clone LN3; eBioscience, San Diego, CA) or with FITC-conjugated anti-canine MHCII (clone YKIX334.2 ThermoFisher Scientific) and with (both canine and human) PE-conjugated anti-human CD86 (clone IT2.2; ThermoFisher Scientific). Cells were incubated with primary antibodies for 30 min protected from light. After washing, cells were analyzed on a Beckman-Coulter Gallios flow cytometer after the addition of 7AAD (ThermoFisher Scientific) to exclude dead cells. Cell analysis was done using FlowJo v9 software (BD Life Sciences, Franklin Lakes, NJ). Comparative expression of MHCII and CD86 was determined by mean fluorescence intensity (MFI) measurements from flow cytometry data.

### TNFα secretion by human and canine macrophages

Following IFNγ treatment, culture supernatants were removed and stored at − 20 °C for analysis of TNFα secretion by ELISA, using the DuoSet® human TNFα ELISA and the DuoSet® canine TNFα kits (R&D Systems, Minneapolis, MN) according to the manufacturer's instructions. Undiluted samples from both canine and human control and stimulated cultures were used for TNFα ELISA. Cell culture media was used for background correction. Below background OD (optical density) levels are represented as zeros on figures.

### RNA sequencing of untreated and PHA-treated PBMC cultures

PBMC from both species were prepared as described above and incubated with PHA (5 ug/ml) for 24h, in 24-well plates in triplicate wells. The RNA sequencing studies used samples from n = 4 dogs and n = 5 humans. For RNA sequencing, the following dogs were included: 6 year-old female poodle, 7 year-old male poodle, 3 year old female mixed breed and 2 year old male mixed breed dog. For human samples, ages ranged from 23 to 60 years old, including 2 females and 3 males. After 24 h incubation, cells were collected and washed twice using sterile PBS. RNA was extracted from cells using Qiagen RNeasy mini kit (Qiagen, Hilden, Germany). RNA concentration was verified on Nanodrop 1000 Spectrophotometer (ThermoFisher Scientific), and then the RNA samples were sent to Novogene Corp. Inc. (Sacramento, CA) for mRNA sequencing. RNA quality was determined using an Agilent 2100 Bioanalyzer system to generate RIN numbers (RNA integrity number), which ranged from 7 to 9.8 for all RNA samples submitted. At Novogene Corp, the mRNA was purified using poly-T oligo-attached magnetic beads. After fragmentation, the first strand cDNA was synthesized using random hexamer primers followed by the second strand cDNA synthesis. The library was completed following end repair, A-tailing, adapter ligation, size selection, amplification, and purification. Quantified libraries were pooled and sequenced on an Illumina NovaSeq 6000 (Illumina, San Diego, CA). 150bp paired end reads were generated, and files were delivered as de-multiplexed fq files. An average of 4.64 e7 reads were obtained for human samples, and an average of 4.52 e7 reads were obtained for canine samples.

Sequence data were analyzed on Partek Flow software, version 10.0 (Partek Inc. Chesterfield, MO). Raw data were filtered by removing reads containing adapters and reads containing N > 10% and for Phred scores > 30. Filtered reads were aligned with STAR 2.7.3a, using either CanFam3.1 genome assembly or GRCh38 human genome assembly. Aligned reads were annotated and counted using HT-seq^21^ with Ensembl 104 for CanFam3.1 and release 107 for GRCh38^22^, differentially expressed genes were identified using DEseq2^23^. Raw reads from either human or canine dataset were normalized separately using CPM method^24^, then normalized reads were combined for matched human and dog genes. Orthologs were detected for canine dataset using biomart and converted to human gene names. Biological interpretations included gene ontology and gene set enrichment analysis (GSEA), which were performed using GSEA (https://www.gsea-msigdb.org/gsea/index.jsp). Gene sets Hallmarks v2022.1, biocarta v2022.1, KEGG v2022.1, Gene Ontology go.bp v2022.1, and ImmuneSigDB v2022.1 were used for comparisons^25^. Significant pathways were filtered using FDR q-value of $\le$ 0.25 and NOM *p*-val $\le$ 0.05, then selected for keyword “immune” or “T_CELL”. Signal2Noise metric was used for ranking gene sets.

### Statistical analysis

Statistical analysis for flow cytometry and ELISA results was performed using GraphPad Prism versions 9.5 and 10.1 (GraphPad Software, San Diego, California USA). Corresponding statistical tests appropriate for each experiment are indicated in Figure legends. Significance for RNA sequencing differential analysis was computed with DEseq2^23^ using Partek Flow software, version 10.0 (Partek Inc. Chesterfield, MO). For gene set and pathway analysis, Signal2Noise metric was in GSEA was used for ranking significant gene sets.

## Results

### Comparison of T cell proliferative responses to PHA activation reveals equivalent responses

**Figure 1:**
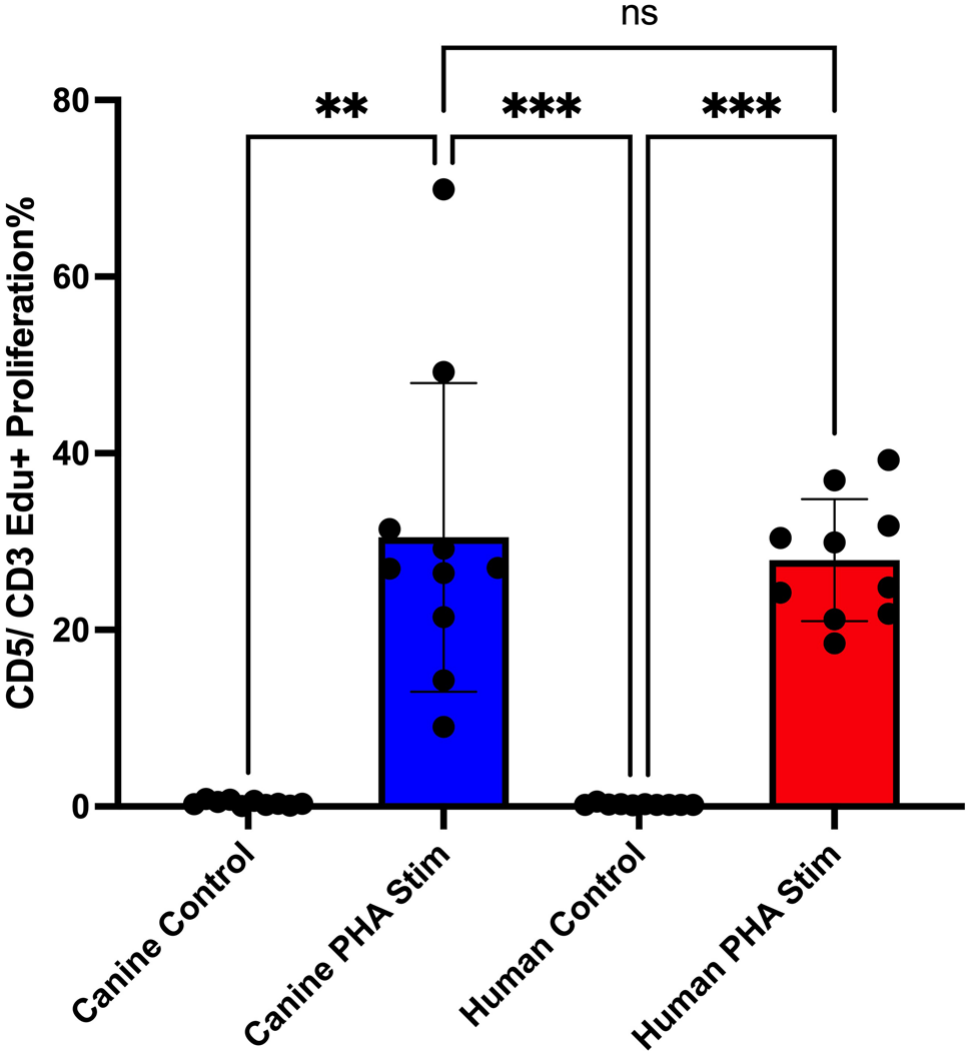
Comparison of proliferative responses of canine and human T cells to PHA activation. Cultures of PBMC obtained from n = 10 healthy dogs (blue) or n = 10 healthy human volunteers (red) were stimulated in triplicate wells for 72h with 5 ug/ml PHA. T cell proliferative responses were measured using incorporation of EDU,and assessed by flow cytometry. In (**A**) the y axis depicts percentage of proliferating T cells, measured as CD5 + cells (canine) or CD3 + cells (human). Statistical significance was performed using GraphPad Prism version 10.1. Statistical significance was determined using non-parametric Kruskal–Wallis test with Dunn’s post-test. Significance denoted as ****p < 0.0001. **p < 0.01, *p < 0.05. was performed using GraphPad Prism version 10.1. Bars depict mean values, with SD (standard deviation). Unstimulated matched cell cultures served as controls.

To assess the suitability of PHA treatment as a means of equivalently activating canine and human T cells, we measured total T cell proliferation (CD5 + T cells in dogs, and CD3 + T cells in humans) by EdU incorporation after 72h of PHA activation of PBMC cultures (Fig. 1). We did not observe significant differences in proliferation between the two species following activation by PHA, thus demonstrating the suitability of PHA an activating agent for T cells from both dogs and humans. On average 30.9% of canine CD5^+^ T cells have EdU incorporation, compared to an average of 27.9% of human CD3^+^ T cells.

### Analysis and comparison of transcriptome responses by canine and human leukocytes following PHA activation of PBMC cultures reveals both similarities and differences

We next employed RNA sequencing to elucidate and compare transcriptional responses to non-specific T cell activation between the two species. Use of transcriptome analysis in this context can provide an in-depth and unbiased comparison of immune responses between the two species. The use of RNA sequencing and transcriptomic analysis also allowed us to circumvent the reagent limitations that can limit the scope of canine immune analyses.

**Figure 2:**
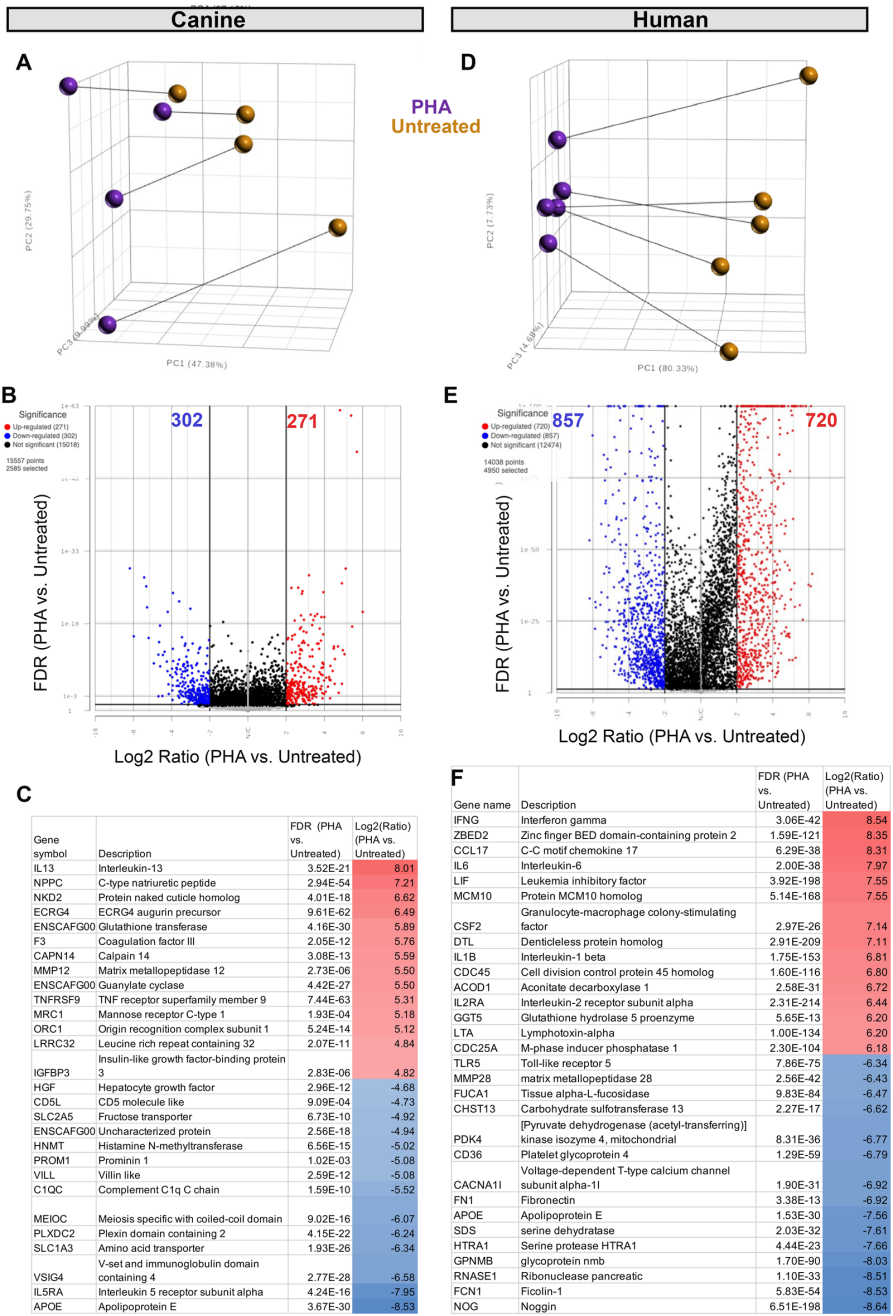
Transcriptomic analysis and comparison of responses of canine and human leukocytes following PHA stimulation. In panel (**A**), principal component analysis (PCA) of PHA-activated vs resting canine PBMC (n = 4, left column) and PHA-activated vs resting human PBMC (**D**, n = 5, right column) depicting variance (similarity) as measured by Euclidean distance. Gold circles depict resting untreated PBMC, while purple circles depict PHA activated PBMC. In (**B**), a volcano plot depicts significantly upregulated genes (red) (log2 fold-change >=2, FDR *p*-value <= 0.05) and significantly downregulated genes (blue, log2 fold-change <= 2, FDR *p*-value <= 0.05) for canine cells (left column), while (**E**) depicts most upregulated and downregulated genes for activated human PMBC (right column). In panel (**C**), heat maps depict the top 15 most-upregulated and top 15 most-downregulated genes in activated canine PBMC, with upregulated genes in red and most downregulated genes in blue. Panel (**F**) depicts most up- and downregulated genes in human activated PBMC cultures.

RNA sequencing was performed on PBMC cultures from n = 4 dogs and n = 5 humans, using triplicate wells of PBMCs activated with PHA or untreated for 24h in culture. (Fig. 2) Samples for analysis were randomly selected from the available donors, since not all samples were subjected to sequencing. PCA (principal component analysis) revealed that PHA activation induced marked transcriptomic changes in cells from both dogs and humans (Fig. 2A and D). The unstimulated and stimulated PBMC transcriptomes of human PBMC displayed less heterogeneity compared to canine PBMC transcriptomes. Canine activated PBMCs had 573 significant differentially expressed genes (DEG) using the parameters of FDR $\le$ 0.05, fold change FC $\le$ − 2 Log2or $\ge$ 2 Log2) (Fig. 2B), when compared to non-activated canine PBMC. In comparison, in activated vs non-activated human PBMC there were 1577 statistically significant DEGs.

We then used the DEGs to interrogate and compare the impact of T cell activation on individual gene expression in the two species (Supplemental Table 1). This analysis revealed that the DEGs were surprisingly distinct between the two species. For example, the most upregulated gene expressed by activated canine PBMC was IL-13 (a prototypical Th2-cytokine), whereas IFN-γ (a prototypical Th1 cytokine) was the most upregulated gene in activated human PBMC cultures. Within the top 20 most upregulated and downregulated genes in dogs and humans, there was also relatively little overlap (Fig. 2C and F).

### Direct comparison of human and canine T cell activation genes reveals important differences

**Figure 3:**
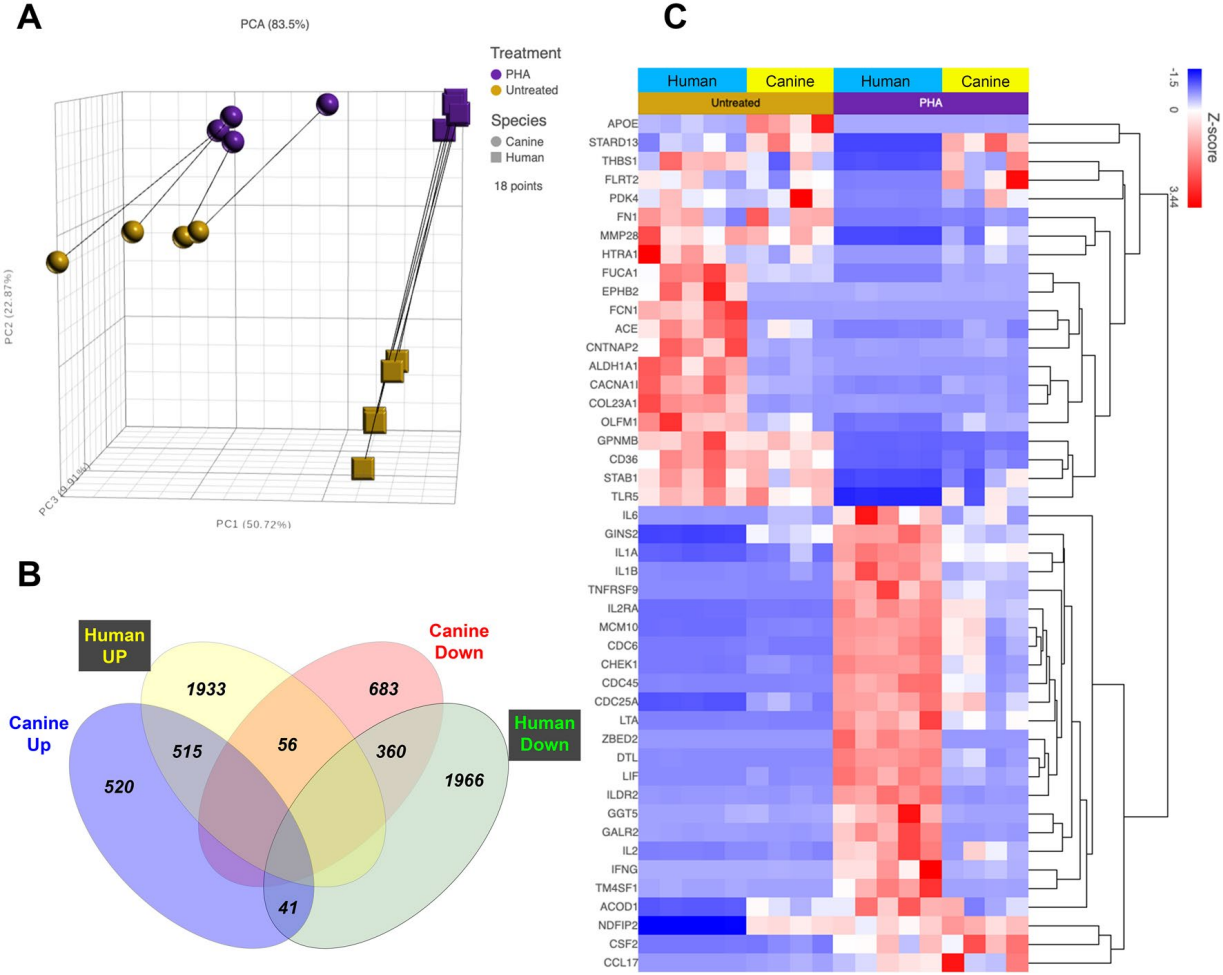
Comparison of canine and human leukocyte transcriptomic responses to PHA activation. Normalized values from a total of 15,326 matched genes in canine and human datasets were used for combined analysis. (**A**), principal component analysis (PCA) is used to depict the relatedness of canine (n = 4, circles) and human (n = 5, squares) leukocyte resting (brown) and PHA-activated (purple) transcriptomes. (**B**) Venn diagrams of overlapping and non-overlapping gene sets from canine and human activated PBMC cultures, as determined by differential gene expression analysis using FDR <  = 0.05. (**C**) heat map of normalized counts for 50 genes including 25 most significantly upregulated in human stimulated PBMC and 25 most significantly down regulated in human PBMC. Species label cyan for human, yellow for canine. Untreated labeled in gold and PHA stimulated in purple.

Next, the normalized counts of human and canine transcripts were used to compare the overall relatedness of gene expression between the two species (Supplemental Table 1**)**. Out of approximately 20,000 protein coding genes with annotations in the canine transcriptome, 15,122 genes were matched exactly to human gene IDs (see GEO submission). An additional 323 canine genes had human gene orthologues (Fig. 3). Combining the normalized counts of shared genes between dogs and humans revealed distinct separation by species based on PCA plot (Fig. 3A), again highlighting the heterogeneity of canine samples, versus the relative homogeneity of the human samples, despite the diversity of ages and nationalities. A Venn diagram (Fig. 3B) was generated to visualize the degree of gene expression overlap between the two species. This analysis highlighted the numbers of overlapping genes between the upregulated and downregulated genes between species, but also illustrated important populations of genes that were not shared (Fig. 3B).

Applying a false discovery rate (FDR) of $\le$ 0.05., there were 515 shared upregulated genes between dogs and humans, and 360 genes that were shared between the two species amongst the significantly downregulated genes. Thus, approximately 50% of all canine DEGs were co-expressed by human activated PBMC, while only approximately 15% of human DEGs were co-expressed in dog PBMC (Fig. 3B).

To help further visualize the similarities and differences in gene expression between dogs and humans in genes related to T cell activation, a heatmap was generated using the top 25 most upregulated and most downregulated genes in human and dog activated PBMC (Fig. 3C**,** Supplemental Fig. 5). From this analysis, it was apparent that expression of many of the most upregulated genes associated with T cell activation in human leukocytes was not similarly upregulated in canine leukocytes (Supplemental Figs. 3, 4). Similarly, many of the most downregulated genes in activated human cells were not changed in canine leukocytes. Thus, the picture that emerged was one of substantial differences between dogs and humans in the terms of the most highly expressed or downregulated genes following T cell activation.

**Figure 4:**
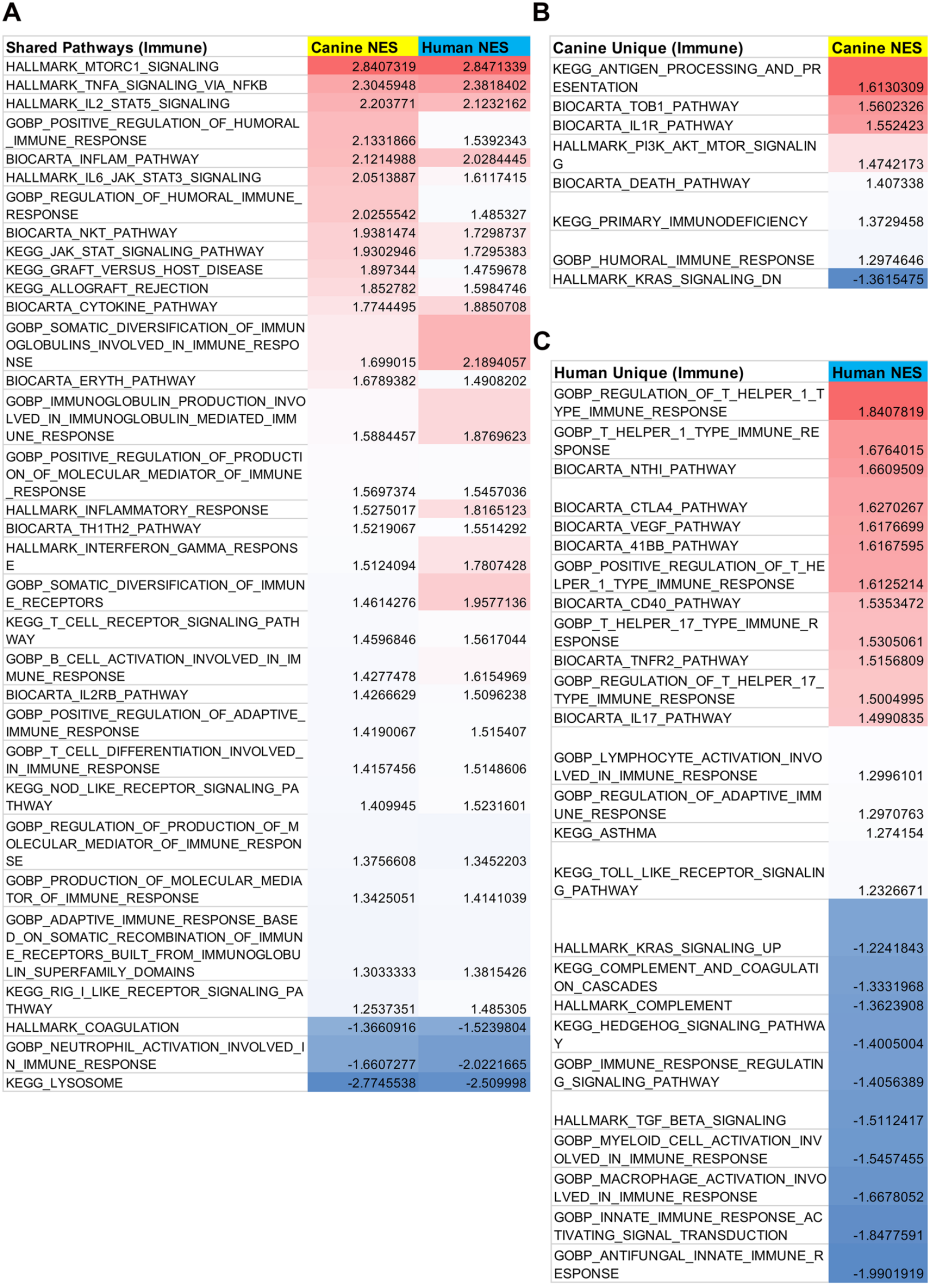
GSEA pathway analysis of PHA stimulated human and canine leukocytes. PBMC cultures of canine and human were activated with PHA and subjected to RNAseq. Pathway analysis using GSEA revealed in (**A**) a total of 33 shared immune related significant pathways in dog versus human immune pathways, out of 106 total possible pathways. The normalized enrichment scores (NES) for each species are depicted in (**B**) and (**C**) (columns 2 and 3). The data are colored red for highest NES scores (positive, 2.84) to blue for lowest NES scores (negative; − 2.77). In (**B**), there were 8 immune related pathways unique to canine cells, with the NES depicted in column 2, with scores color labeled from high to low. In (**C**), there were 26 significant immune related pathways unique to human cells. The NES scores are depicted in column 2.

Finally, we used gene set enrichment analysis (GSEA) to better understand and compare pathways used by activated canine and human T cells (Supplemental Fig. 2**)**. This analysis revealed many shared pathways between canine and human activated PBMC, with a total of 106 significant (p value <= 0.05) immune related pathways found. Out of the 106 immune or T cell related pathways, 33 were shared between species, with matched upregulated or downregulated scores overall. (Fig. 4A). In contrast, there were 8 immune-related pathways that were found to be unique to canine activated PBMC, and these included antigen processing, cell death, humoral immune response pathways (Fig. 4B). There were also 26 unique immune pathways in the human dataset (Fig. 4C), which notably included Th1 and TH17 related pathways, as well as pathways related to cytotoxic T cells. Several complement pathways were also exclusively downregulated in human PBMCs. Overall, the pathway analyses revealed many shared immune pathways between dogs and humans, but also notable differences, including in particular the divergence of Th1 and Th17 pathways and antigen presenting cell pathways.

### Canine T cells demonstrate reduced capacity to produce inflammatory cytokine upon activation

**Figure 5:**
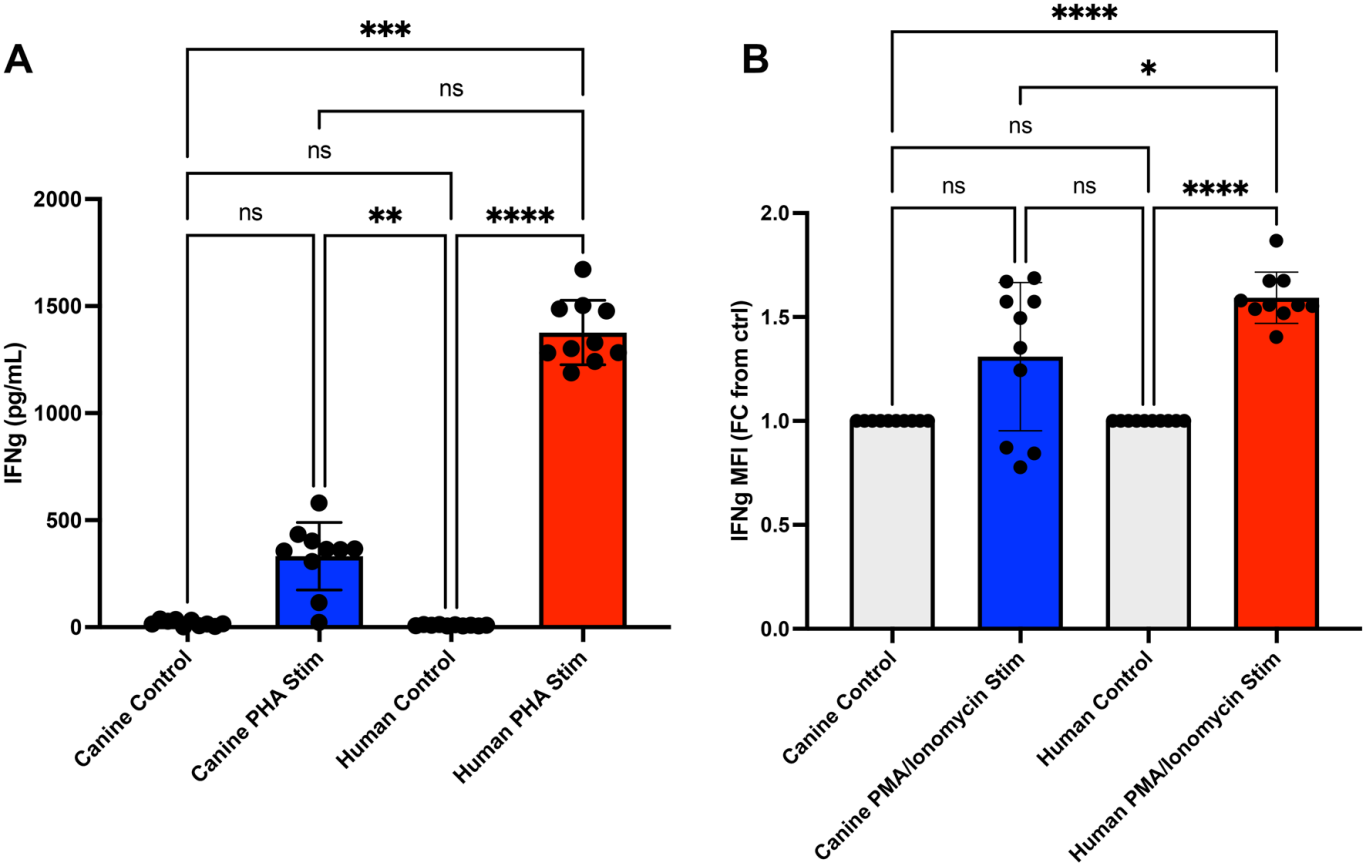
Assessment of relative capacity to product IFN-g by T cells activated in vitro with PHA or PMA/ionomycin, in PBMC cultures from dogs and humans. In (**A**), the concentrations of IFN-g (pg/ml) secreted by PHA activated PBMC cultures for dogs and human were measured by ELISA. In (**B**) the fold change (FC) of the geometric mean fluorescence intensity for PMA/ionomycin activated versus non-activated cells is depicted on y axis. Statistical analysis was performed using GraphPad Prism version 10.1. Statistical significance was determined using non-parametric Kruskal–Wallis test with either (**A**) Dunn’s multiple comparisons post hoc test, or (**B**) uncorrected Dunn’s test for normalized values. Significance denoted as ****p < 0.0001. **p < 0.01, *p < 0.05. Bars depict mean with SD (standard deviation).

To validate one of the key transcriptomic differences identified between human and canine T cells revealed by RNA sequencing, we measured IFN-γ production following PHA stimulation of PBMC cultures (Fig. 5A). Following activation with PHA, secretion of IFN-γ was increased in both species. However, the relative amount of IFN-γ produced by activated canine T cells was numerically much less than that produced by activated human T cells (fourfold difference), though the difference was not statistically significant. For example, the mean IFN-γ concentration produced in PHA activated dog PBMC cultures was 331 pg/ml, compared with 1377 pg/ml for activated human PBMC cultures. These results are therefore in agreement with the transcriptomic analyses and indicate that innon-specific activation of T cells with PHA produced fewer overall changes in gene expression.

It was possible the differences observed between canine and human IFN-γ secretion were influenced by the T cell stimulus employed, namely the use of PHA as a non-specific T cell mitogen. To address this, we also employed a different T cell activating stimulus, using the combination of PMA and ionomycin, which activates the protein kinase C pathway and thereby bypasses the T cell receptor directed pathway of T cell activation (Fig. 5B**,** supplemental Fig. 5) ^26^. Using PMA/ionomycin activation, canine T cells also produced significantly less IFN-γ intracellularly than human T cells (Fig. 5B). Thus, T cells from dogs had a significantly reduced capacity to produce IFN-γ compared to T cells from humans.

### Canine macrophages show enhanced sensitivity to IFN-γ activation

To better understand the significance of reduced IFN-γ production by canine T cells, we next investigated the relative sensitivity of canine and human antigen presenting cells (macrophages) to IFN-γ activation. This property is also important, as not only the amount of IFN-γ produced, but also the sensitivity of target cells to IFN-γ signaling determines the magnitude of inflammatory responses^27^. IFN-γ is known to be a key cytokine regulating activation of macrophages and DC, and their ability to control pathogens and present antigens to T cells^28^. Canine and human macrophage cultures (n = 4 cultures for each species were analyzed, and selected randomly from available donors) were then exposed to varying concentrations of recombinant IFN-γ (species-specific) and responses to IFN-γ activation were assessed, including upregulated expression of the co-stimulatory molecules MHCII and CD86, and secretion of TNF-α^29^.

**Figure 6:**
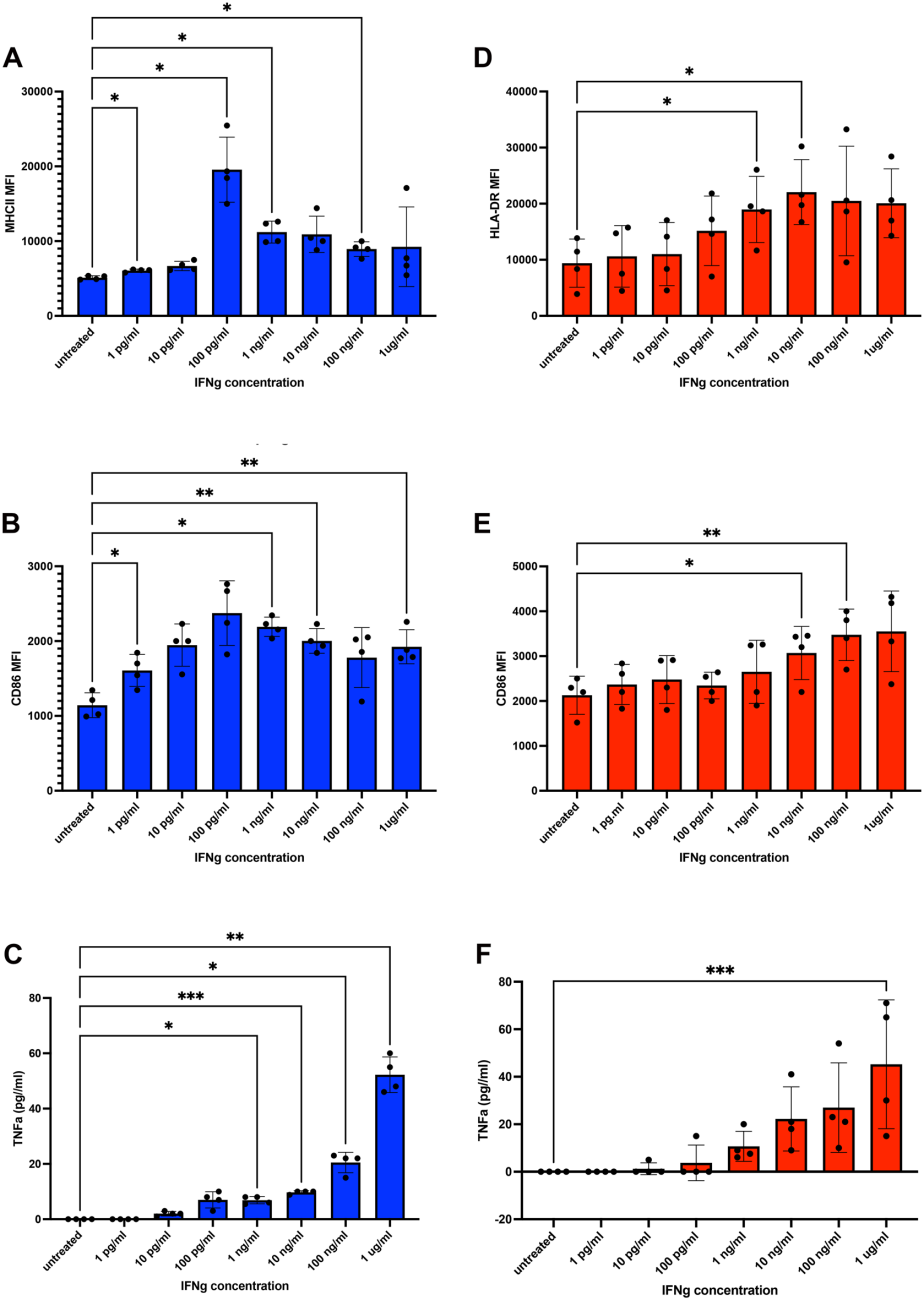
Comparison of macrophage responses to in vitro treatment with IFN-g. Cultures of n = 4 human (red bars) or n = 4 canine (blue bars) monocyte-derived macrophages (MDM) were treated with increasing dosages of recombinant human or canine IFN-g, respectively, for 36 h. Bars depict mean with SD (standard deviation). In (**A**) and (**D**) the impact of IFN-g treatment on expression of MHCII molecules on the surface of MDM as assessed by flow cytometry is displayed, as described in Methods. The geometric mean expression (MFI) is depicted on y axis, with increasing concentrations of IFN-g displayed on the x-axis. In (**B**) and (**E**) expression of the co-stimulatory molecule CD86 and the impact of increasing concentrations of IFN-g is depicted. Statistical analysis was done using paired repeated measures one way ANOVA with Bonferroni multiple means comparison test applied. *p < 0.05, **p < .01 and ***p < 0.001. In (**C**) and (**F**) supernatants were obtained from MDM cultures after 48h of culture in the indicated concentrations of IFN-g. and assayed for TNF-a release by ELISA. Statistical analysis was done using non-parametric Friedman test for repeated measures with post hoc Dunn’s multiple comparisons test. *p < 0.05, **p < .01 and ***p < 0.001.

Importantly, we observed that canine macrophages were significantly more sensitive to IFN-γ activation than human macrophages. For example, the concentration of IFN-γ required to stimulate significant upregulation of MHCII expression by canine macrophages was 100 pg/ml (Fig. 6A), compared to 1 ng/ml of IFN-γ for human macrophages (Fig. 6D). Significant upregulation of CD86 expression by canine macrophages occurred at an IFN-γ concentration of 1 pg/ml (Fig. 6B), as opposed to 10 ng/ml (Fig. 6E) to trigger significant upregulation of CD86 by human macrophages. Thus, costimulatory molecule upregulation by canine macrophages was much more sensitive to low concentrations of IFN-γ compared to human macrophages.

We also quantitated secretion of TNF-α by macrophages following IFN-γ stimulation. Similar macrophage IFN-γ sensitivity differences were observed, with at low stimulation doses of 10pg/mL and 100 pg/mL IFN-γ, canine macrophages produced an average of 2 ~ 7 pg/mL of TNF-α, whereas human macrophages only secreted an average of 1.25 ~ 3.75 pg/mL TNF-α (Fig. 6C and F). These findings therefore indicate that macrophages in dogs were more sensitive to activation by IFN-γ, which may represent a compensatory mechanism for responding to lower amounts of IFN-γ produced by activated canine T cells.

## Discussion

The studies reported here are amongst the first to our knowledge to comprehensively compare human and canine immune responses to classical activating stimuli. Our studies employed RNA sequencing and functional assays to compare how T cells and macrophages from the two species responded to activation. We found that in general, the trends in canine and human T responses to activation were similar, as assessed by GSEA and pathway analysis (Figs. 3 and 4). There were however also notable differences, particularly with respect to the Th bias of immune responses, with human activated cells expressing a notable Th1 bias while T cells from dogs lacked a uniform Th1 signature and exhibited some Th2 signatures (Supplemental Fig. 2). However, as transcriptomic studies become more widespread it is increasingly recognized that the functional classification of Th1 and Th2 T cells may be an oversimplification^30,31^. Indeed, looking at individual gene expression for Th1 genes such as TBX21, RORC, GZMB, IL21 (Supplemental Fig. 3) it can be appreciated that both activated canine T cells human T cells exhibit increased expression of these genes. Conversely canine IFNG, CTLA4, TNF, IL1B genes did not demonstrate the same dramatic upregulation in expression exhibited by human activated PBMCs. Nevertheless, after integration with larger datasets (Supplemental Fig. 2), the T cell activation status of canine T cells shows less clear-cut Th1 or Th2 bias and the lack of differential expression of Th1 genes in canine T cells may results in part due to higher basal expression of Th1 genes by human T cells.

Additionally, T cells from dogs exhibited an overall reduced transcriptomic response to PHA activation, with the normalized expression values of nearly all genes being noticeably reduced in dogs compared to humans. Functional analysis of T cells (IFN-γ secretion) and antigen presenting cells (co-stimulatory molecule expression, TNF-α secretion) were largely concordant with the results of the transcriptomic analysis. Thus, the overall picture that emerges is one in which canine T cell responses are subdued relative to human T cells, and without the inherent Th1 bias evident in human T cells, though still sharing many of the same immune activation pathways. The greater sensitivity of canine macrophages to activation by IFN-γ also suggests a possible compensatory mechanism for responding to the lower amounts of IFN-γ produced by canine T cells and NK cells.

In recent years, there has been increasing interest in the use of dogs with spontaneous cancer as a more clinically relevant large animal model for cancer immunotherapy research. Indeed, the value of the dog cancer model was recognized by the Institutes of Medicine, and substantial new funding has been allocated to canine cancer immunotherapy research^1,2,32^. There are several notable examples of the canine cancer model being used to help develop human cancer immunotherapies, including muramyl tripeptide for osteosarcoma, xenogeneic cancer vaccines for melanoma, and checkpoint antibodies for invasive bladder cancer^6,15,33^. Our group has also reported recently the TME modifying immunotherapies using repurposed drugs for treatment of canine metastatic osteosarcoma and brain cancer^8,10^. In the case of the canine osteosarcoma studies, the findings helped initiate a very similar trial in pediatric metastatic osteosarcoma patients (NCT03900793 clinicaltrials.gov).

Despite the interest in the dog as a translational model for cancer immunotherapy research, there have been few studies directly comparing immune responses between dogs and humans^34,35^. Genetically, dogs and humans are relatively closely related compared to humans and mice^36^. The relative numbers of circulating immune cells are quite similar in dogs and man^17^, but their functionality has not been investigated systematically. In a recent study comparing canine NK cells to those of humans and mice, Gingrich et al. observed that canine and human NK cells were more alike than mouse and human NK cells, but also noted that the transcriptomes of dog and human NK cells were distinct^35^. One important finding from their studies was that canine NK cells exhibited significantly reduced expression of IFN-γ transcripts, along with reduced expression of effector molecules such as Granzyme B, compared to human NK cells. Similarly, we recently investigated the transcriptomes of in vitro polarized canine macrophages, and found little overlap between the transcripts of polarized macrophages from dogs and humans^37^.

Our studies here report important shared immune pathways between dogs and humans, including the TNF-α pathway, IL-2 and IL-6 pathways, T cell receptor signaling pathways, and RIG-I pathways (Fig. 4). This degree of conservation of responses is not surprising given the genetic relatedness of dogs and humans^36^. However, we were also surprised to discover significant and potentially meaningful differences in the activated immune transcriptome pathways. The observed differences could stem from unique features of the T cell signaling pathways in the two species, and this cannot be ruled out in our studies. However, arguing against this is the fact that canine T cells also produced significantly less IFN-γ following activation by a T cell receptor independent pathway, using PMA/ionomycin activation (Fig. 5). A more likely explanation is that differences in T cell responses observed here may reflect inherent differences in T cell cytokine bias^38^. Our findings with canine T cells are also in agreement with canine and human NK cell comparative studies, where it was observed that there was significantly reduced expression of IFN-γ and related genes such as GZM by canine NK cells, compared to human NK cells^35^.

There are several caveats to the studies reported here. For one, samples from relatively few individuals of each species were evaluated, thus limiting the biological sample size. This was compensated to some degree by including a reasonable diversity of ages and genders in the study to minimize this sample size effect. Animal to animal sample variability was also much more pronounced for dogs than for human samples. This variability would have in turn reduced the number of DEGs and the number of significant changes that were detected in canine samples. In addition, pathway analysis in species other than mouse and humans is more difficult to perform, since the data used to generate such pathways are derived entirely from human and mouse samples. The overall transcript abundance was also much lower in dogs compared to humans.

Transcriptomic analysis was performed on PBMCs treated for 24 h in an effort to standardize the activation status; nevertheless it remains possible that the species differences may have normalized over longer time in culture (eg, culture for > 3 days). Future studies may include time course experiments coupled with enrichment of T cells and different biologically relevant stimuli such as bacterial superantigens to explore the full spectrum of differences between dog and human T cell activation responses. Concerning the transcriptome analysis pipeline, although there is precedence for the direct comparison of RNA sequencing data from different species^39,40^ differences in normalization, alignment and gene annotations must also be taken into account when directly comparing expression counts^41^. For the direct comparison of human and dog transcripts, we also performed a combined normalization using common CPM (counts per million) median ratio to help address these effects. Although the use of this approach changed the “counts” of each gene, the scale of the gene expression changes remained relatively unchanged and did not change the overall conclusions from the data presented in Fig. 3.

The macrophage response assays were intended as a functional readout to explore the differences in biological responses to a key inflammatory and immune regulatory stimulatory cytokine (IFN-γ). However, there currently is no standardized method for determining the biological activity of IFN-γ across species^42,43^, such that it is possible that the in vitro activity of recombinant IFN-γ used in these studies differed between dog and human IFN-γ despite the identical amount of cytokine protein used. Reagent availability for dogs can also be a limitation for immunological assays^44^. Thus, RNA sequencing is an ideal method to circumvent these reagent limitations since it does not require species specific reagents. For the functional assays described in this manuscript, we used validated antibodies and proteins from reputable companies that have been used in many previous studies. Despite these limitations, the assays utilized in the current study provided an important point of reference for directly comparing immune responses between the two species and provided a baseline for future studies comparing the two species.

The findings from this study may have several implications for cancer immunotherapy research in dogs. For one, the reduced proinflammatory signature, particularly reduced IFN-γ expression by activated T cells and NK in dogs, may reduce the likelihood of dogs experiencing adverse immunological reactions (eg, cytokine release syndrome; CRS) to immunotherapies such as immune checkpoint inhibitors (ICI) or CAR T cells^45,46^. Indeed, there have been no reported examples of CRS in dogs treated with ICI to date, including PD-1 and PD-L1 antibodies^33,47^. Similarly, other than one reported case of mild CRS in a CD20 CAR T cell-treated dog^48^, dogs treated with CAR T cells to date have not experienced significant CRS^49,50^. In contrast, CRS occurs in as many as 70% of human patients treated with ICI or CAR T cells^51–53^. The reduced T cell response to activation in dogs also suggests that ICI or CAR T cell therapies may be less effective in dogs than in humans, since the activity of ICI and CAR T cells relies in part on induction of key effector T cell cytokines such as IFN-γ^54^.

The findings reported here are important for providing context for assessing immune responses in dogs with cancer. Despite the differences in immune responses noted, the results do not however diminish the value of dogs with spontaneous cancers as models for human cancer research, which has been validated over years of study. Rather, the immunological differences can provide guidance in designing immunotherapy trials and selecting relevant immunological biomarker endpoints for trials in dogs with cancer.

### Supplementary Information

Supplementary Tables.

Supplementary Figures.

## Data Availability

The RNA sequencing data including fasq files and read counts presented in this manuscript have been deposited in NCBI's Gene Expression Omnibus and are accessible through GEO Series accession number GSE226767. All other data is available from the corresponding author on reasonable request.
