# Supplementary material for: Direct comparison of canine and human immune responses using transcriptomic and functional analyses
Source: Sci Rep. 2024 Jan 26;14:2207. doi: 10.1038/s41598-023-50340-9 (PMC10811214; doi:10.1038/s41598-023-50340-9)
Supplement: Supplementary file 2 — Supplementary Figures. [file 41598_2023_50340_MOESM2_ESM.pdf]

Supplemental Figure1

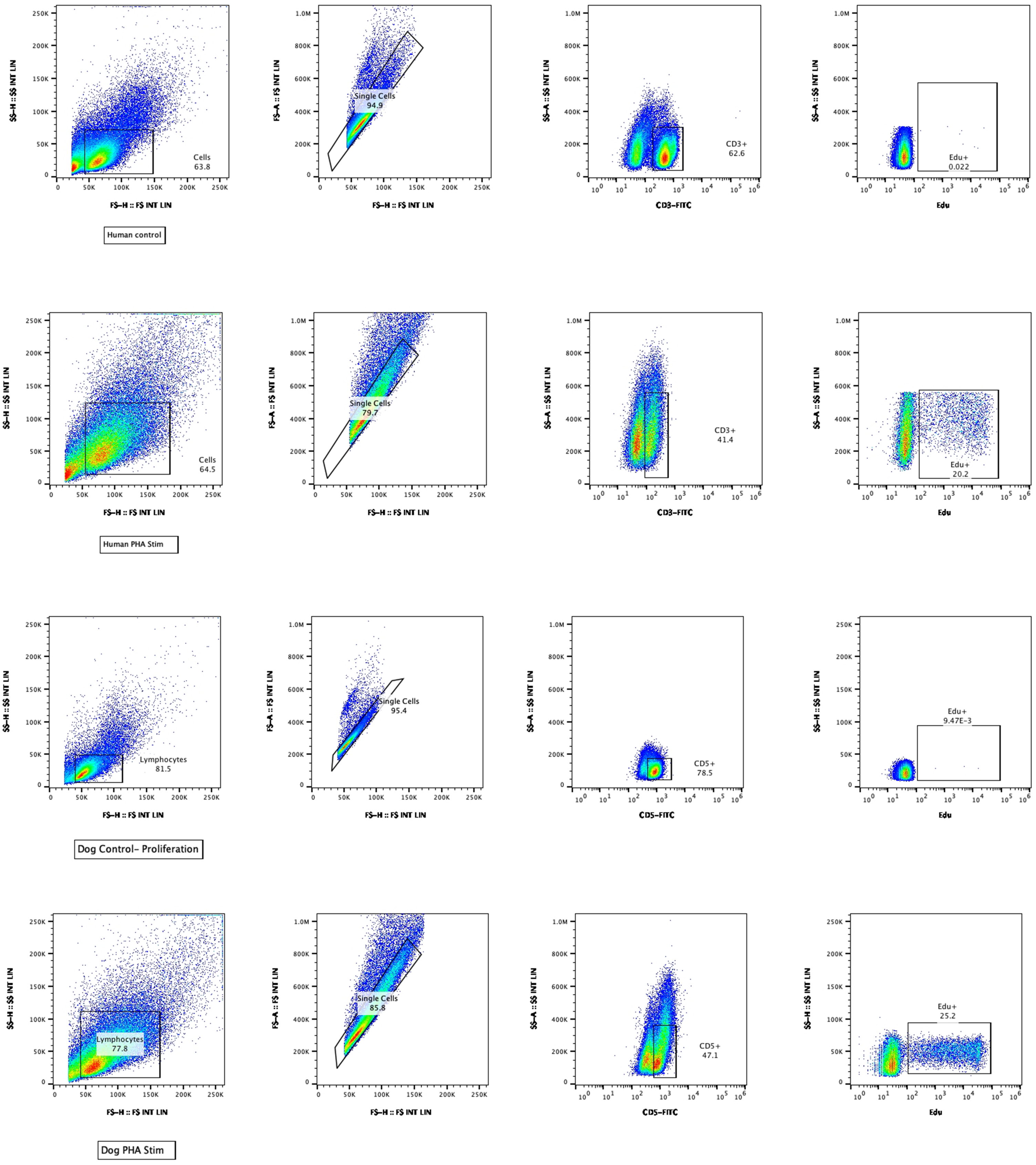

Supplemental Figure 1. Gating scheme for T cell proliferation using Flowjo. Examples of dot plots from LMD files generated from the Gallios flow cytometer, and analyzed using FlowJo. From left to right box shows first the gating of total cells using forward scatter (FS) and side scatter (SS). Then single cell gating to eliminate doublets or clusters, followed by either CD5-FITC or CD3-FITC positive T cell gating. The last box shows Edu+ proliferation as a histogram after gating on T cells. A) Unstimulated canine PBMC control. B) PHA simulated canine PBMC. C) Unstimulated human control. D) PHA simulated human PBMC.

Supplemental Figure2

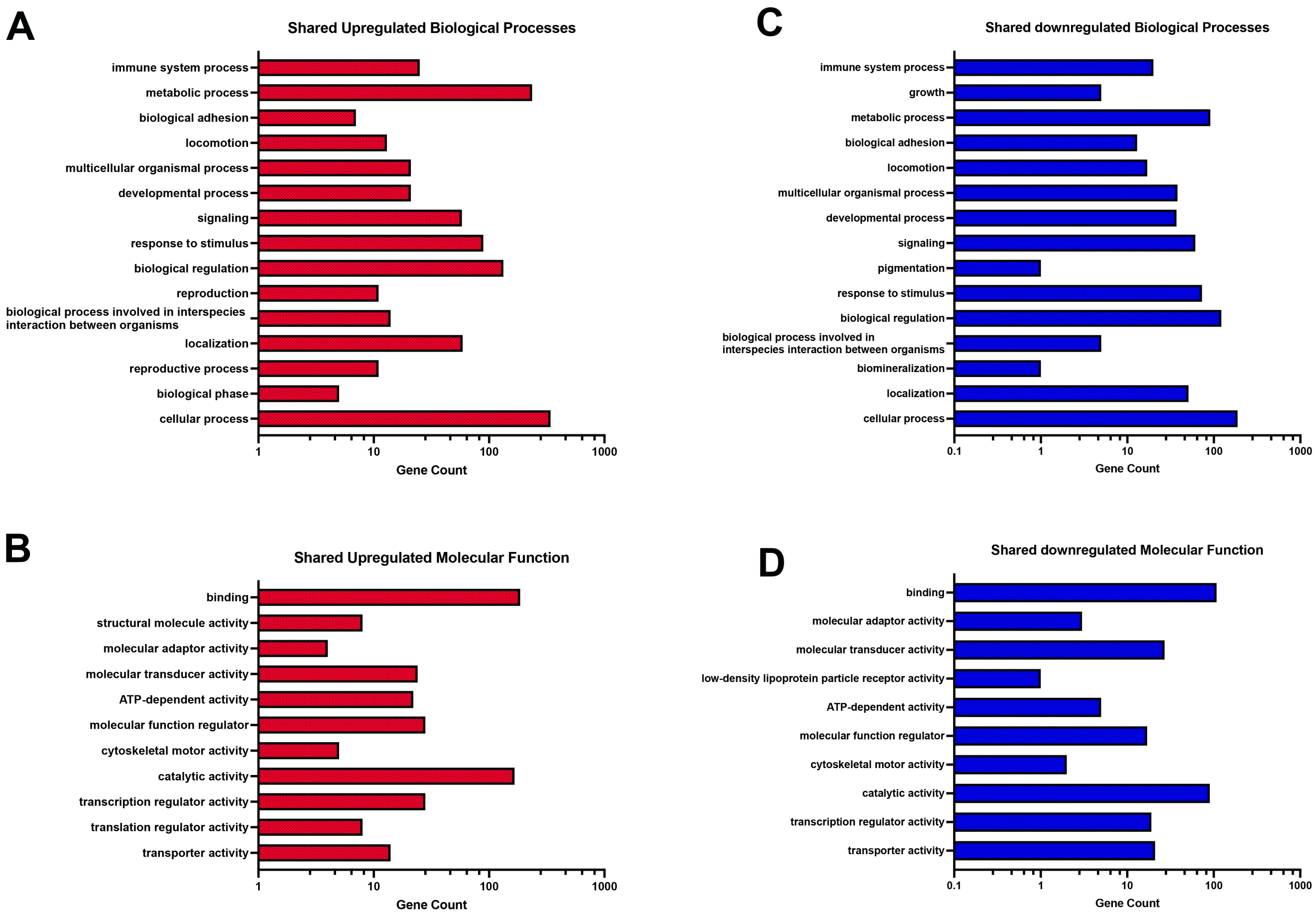

Supplemental Figure 2. Analysis of shared expressed genes for canine in human activated PBMC transcriptome data, using Panther biological process and molecular function grouping. Overlapping gene set between canine and human PBMCs from VENN diagram were selected and analyzed for biological processes. A total of 515 shared upregulated genes or 360 shared downregulated genes were used for categorization. A) bar graph shows 15 categories of biological processes belonging to shared 515 upregulated genes using human gene annotations. Number of genes for each process shown on x axis. B) bar graph (blue) of 15 biological processes for 360 shared downregulated genes. C) 11 categories of molecular function for 515 shared upregulated genes in red bar graph. D) 10 categories of molecular function for 360 shared downregulated genes. Uncharacterized genes are all removed from graph.

proteins co-occuring with the tissue Th1/Th2 cell in abstracts of biomedical publications from the TISSUES Text-mining Tissue Protein Expression Evidence Scores dataset.

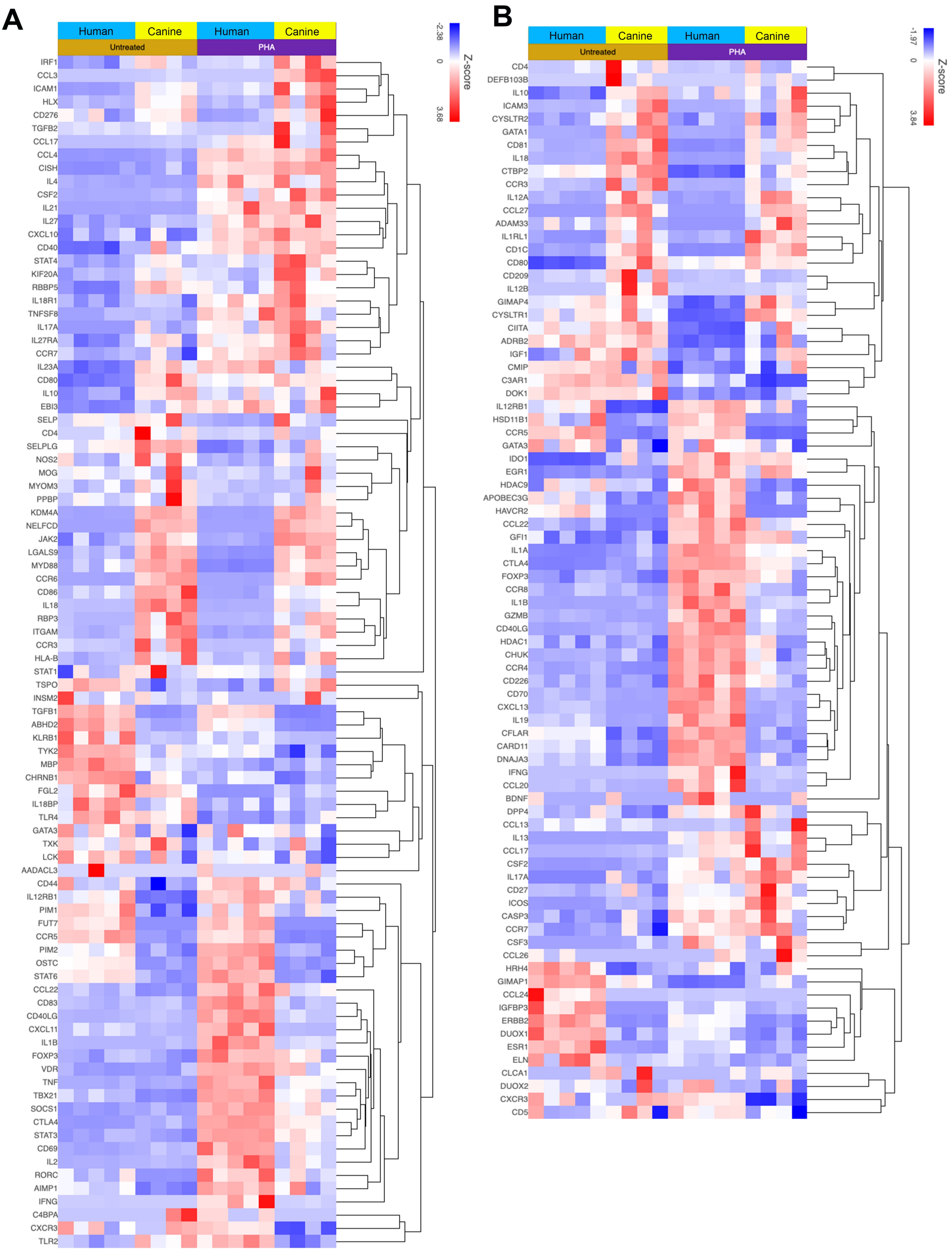

Supplemental Figure 3

Supplemental figure 3: Th1 and Th2 gene expression in dog and human samples. Gene lists were mined from online repository compiled by Ma'ayan Laboratory of Computational Systems Biology. Using the keywords TH1 or TH2. Gene lists were used in combined datasets of canine and human transcriptome to generate heatmap. A) Th1 gene list of 90 genes with expression represented from red (high) to low (blue). Species and treatment labels on top row of heatmap. B) Heatmap of Th2 gene expression of 81 genes.

Supplemental Figure 4

Top 25 upregulated or downregulated genes from canine DEseq results

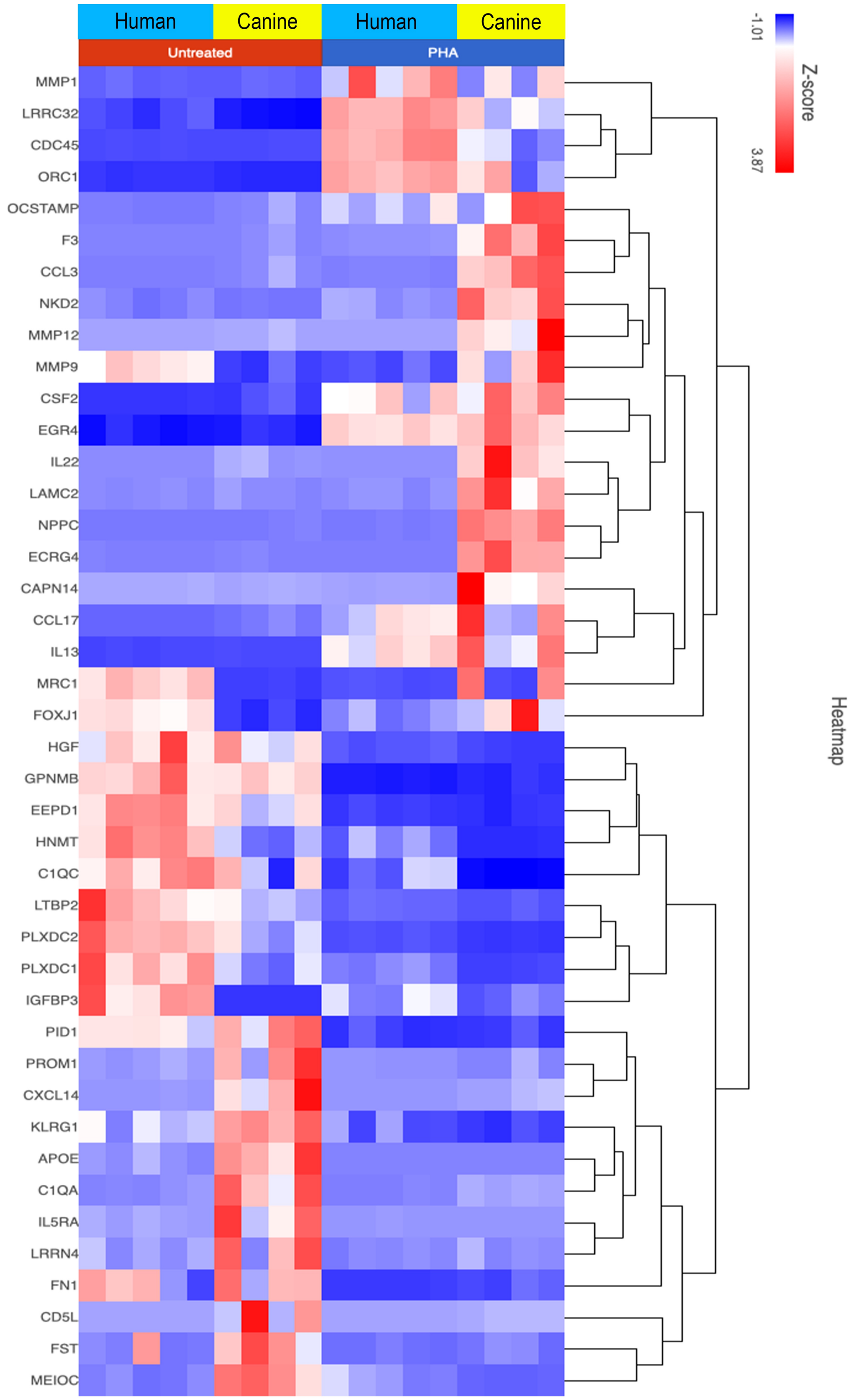

Supplemental figure 4: Heat map and hierarchical clustering of top 25 upregulated and top25 downregulated genes in the canine DEseq comparison results. DEseq results from canine PHA stimulated vs. unstimulated control were used to generate heatmap of human and canine gene expression values. Total gene list of 50 genes with expression represented from red (high) to low (blue).

Supplemental Figure 5

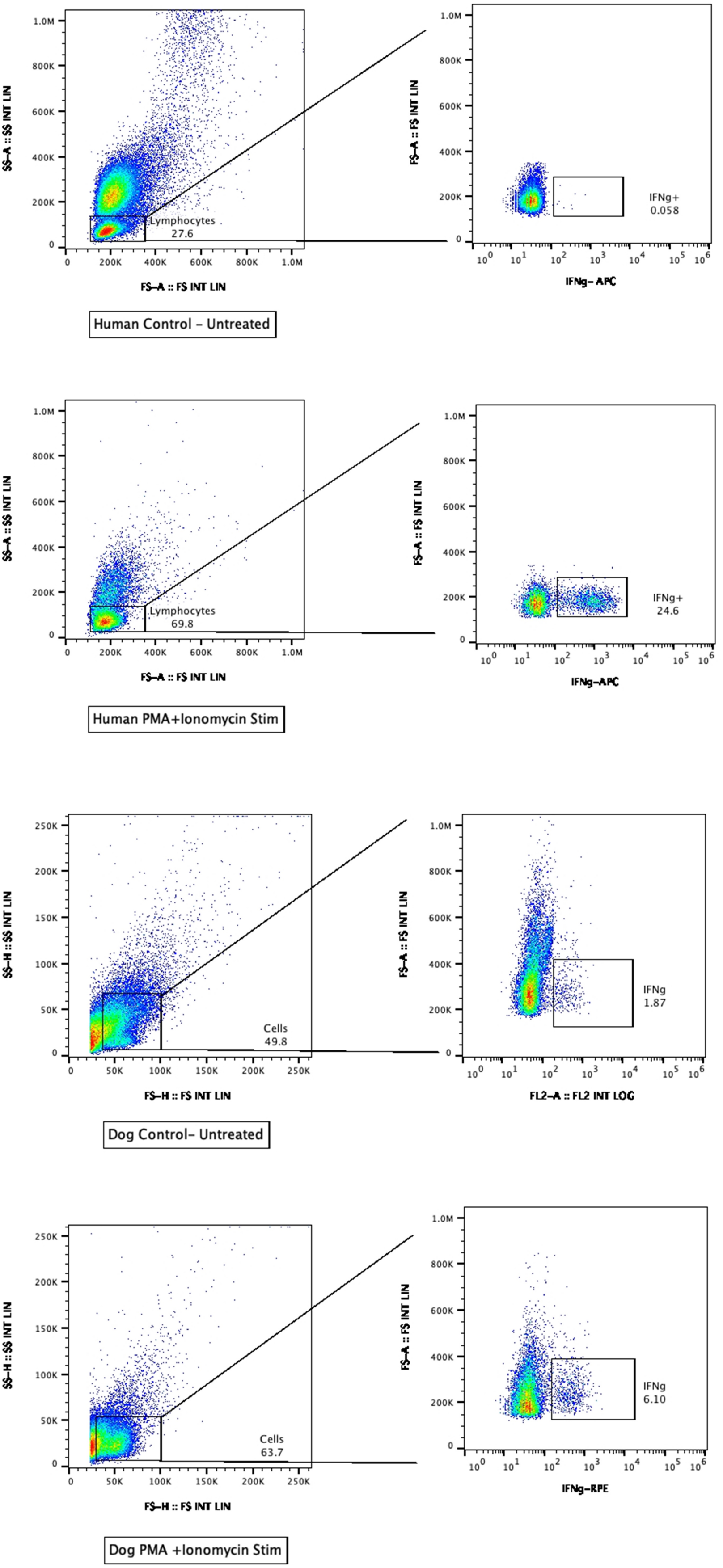

Supplemental Figure 5: Flowjo dot plot of intracellular IFNg staining on canine and human cells after PMC and Ionomycin stimulation. Boxes left to right show cell gating on FS/SS, followed by IFNg positive inset box gate on the right. X axis represents florescent intensity for IFNg-APC and y axis for side scatter.

Supplemental Table 1: Complete list of protein coding genes from canine and human data set. Sheet 1 lists all protein coding genes with description and DESeq results as described in methods. FDR adjusted p value and Log2 fold change (PHA vs. Untreated) in columns 3 and 4. Sheet 2 lists all human protein coding genes with description, DESeq results with FDR adjusted p value and Log2 fold change (PHA vs. untreated). Highest (positive) fold change in red and lowest (negative) fold change in blue.
